# Acoustic monitoring of laser-induced phase transitions in minerals: implication for Mars exploration with SuperCam

**DOI:** 10.1038/s41598-021-03315-7

**Published:** 2021-12-15

**Authors:** Baptiste Chide, Olivier Beyssac, Michel Gauthier, Karim Benzerara, Imène Estève, Jean-Claude Boulliard, Sylvestre Maurice, Roger C. Wiens

**Affiliations:** 1 IRAP-CNRS, Université Toulouse III, 31400 Toulouse, France; 2 Sorbonne Université, Muséum National d’Histoire Naturelle, CNRS UMR 7590, IMPMC, 75005 Paris, France; 3 Los Alamos National Laboratory, Los Alamos, NM United States

**Keywords:** Mineralogy, Laser-produced plasmas

## Abstract

The SuperCam instrument suite onboard the Mars 2020 Perseverance rover uses the laser-induced breakdown spectroscopy (LIBS) technique to determine the elemental composition of rocks and soils of the Mars surface. It is associated with a microphone to retrieve the physical properties of the ablated targets when listening to the laser-induced acoustic signal. In this study, we report the monitoring of laser-induced mineral phase transitions in acoustic data. Sound data recorded during the laser ablation of hematite, goethite and diamond showed a sharp increase of the acoustic signal amplitude over the first laser shots. Analyses of the laser-induced craters with Raman spectroscopy and scanning electron microscopy indicate that both hematite and goethite have been transformed into magnetite and that diamond has been transformed into amorphous-like carbon over the first laser shots. It is shown that these transitions are the root cause of the increase in acoustic signal, likely due to a change in target’s physical properties as the material is transformed. These results give insights into the influence of the target’s optical and thermal properties over the acoustic signal. But most importantly, in the context of the Mars surface exploration with SuperCam, as this behavior occurs only for specific phases, it demonstrates that the microphone data may help discriminating mineral phases whereas LIBS data only have limited capabilities.

## Introduction

Laser-Induced Breakdown Spectroscopy (LIBS) is an analytical technique that relies on the spectral analysis of the photons emitted by a plasma plume excited by a short and energetic laser pulse focused on a solid target. The spectral analysis of the plasma light allows to identify the atoms that compose the ablated matter^1^. Quantification of elemental chemistry may be achieved for major and some minor elements after careful calibration of the instrument. The breakdown process occurs when the laser heats, melts then vaporizes the matter. Therefore, LIBS is destructive and any molecular and mineralogical structure information is lost during the ablation process. Thereby, LIBS is not able to discriminate between two different mineralogical phases with the same chemical composition but with a different structure such as mineral polymorphs. When performed under a surrounding gas, i.e. not under vacuum, the plasma expansion generates a shock wave that relaxes into an acoustic wave. This wave carries rich information that can be recorded and analyzed by a microphone^2^. The energy released within the shock wave actually depends on several target properties such as the optical absorption and the thermal dissipation^3,4^. Moreover, recording the laser sparks has been shown to provide data complementary to the LIBS optical spectrum and key information such as the target’s hardness and the volume of the laser-induced ablation cavity^5,6^.

On February 18, 2021 NASA’s Perseverance rover landed in Jezero Crater, Mars, carrying onboard the SuperCam instrument suite^7,8^, that combines color imaging, LIBS, time-resolved Raman spectroscopy and visible and near-infrared reflectance spectroscopy. This remote sensing instrument includes a microphone used to complement the LIBS investigation of SuperCam, and to record the atmospheric noise in the audible bandwidth^9^. Perseverance is exploring the geology of the Jezero crater and one main scientific objective is to detect possible traces of ancient life on Mars^10^. Perseverance will also cache the first samples for return to Earth by future missions. In that context, SuperCam provides key chemical and mineralogical information on the rocks for in situ science and to optimize the selection of the samples.

To perform remote LIBS analysis of surrounding rocks up to 7 m from the rover, SuperCam uses a telescope to focus the high-irradiance laser and collect the signal subsequently analyzed by a spectrometer. Irradiance deposited on the target ($>1 \ \hbox {GWcm}^{-2}$ over a $\sim \ 300 \, \upmu \mathrm{m}$ diameter spot for SuperCam) creates an ablation crater, up to few hundreds micrometers deep for a 30 shot burst^8^. It may also alter locally the mineralogical structure within this laser-induced crater^11,12^. For example, some high optical absorption minerals have been shown to exhibit laser-induced mineral phase transitions due to melting followed by recrystallization (hematite is transformed to magnetite for instance) or amorphization (graphite into amorphous carbon) within the LIBS crater^11^. Such transitions were completely achieved after 30 shots, for a laser energy comparable to SuperCam. The formation of a new mineral phase or material inside the laser-induced crater has strong implications as the targeted spot may have a different chemical composition as well as new physical properties. Hence, it likely induces some changes in the laser-matter coupling. Consequently, one could wonder whether this change may lead to a modification of the acoustic signal during the shots when the transition takes place.

The goal of this study is to investigate whether an acoustic monitoring of LIBS on some specific mineral phases can highlight some phase transitions and/or transformations induced by the laser ablation. To do this, iron oxides were selected to test this hypothesis: hematite ($\hbox {Fe}_2\hbox {O}_3$), magnetite ($\hbox {Fe}_3\hbox {O}_4$) and goethite (FeO(OH)), owing to their relevance for the exploration of the surface of Mars^13,14^. Moreover, a laser-induced phase transition was previously observed for hematite^11,12^. In addition, a natural diamond and graphite were also selected for their specific physical properties: low (transparency) versus high (opacity) optical absorption of the LIBS infrared laser beam respectively, and extreme hardness for diamond. Moreover, diamonds might eventually be found in Jezero crater due to impact shock metamorphism of carbonates in the margin of the crater. Indeed, diamonds are found in Ureilites meteorites, likely formed due to impact shock^15^.

The minerals were first analyzed with LIBS with acoustic monitoring using the Mars-atmosphere LIBS acoustic calibration test bench^5,6^ equipped with a martian chamber (i.e. using a gas mixture of 95.7% $\hbox {CO}_2$, 2.7% $\hbox {N}_2$, and 1.6% Ar, at a pressure of 7 mbar) at the Institut de Recherche en Astrophysique et Planétologie (IRAP, Toulouse, France). This setup was used to ablate the samples and to record the associated laser-induced acoustic wave. Bursts of 30 laser shots were performed on each sample. In order to study precisely the evolution over the first shots, bursts of 1 to 5 shots were fired on hematite and goethite. In the case of diamond, only an additional point of 3 shots was fired. Subsequently, these craters generated with few laser shots were characterized with Raman spectroscopy including hyperspectral Raman mapping and scanning electron microscopy (SEM) at the Institut de Minéralogie, de Physique des Matériaux et de Cosmochimie (IMPMC, Paris). A detailed description of the instruments and the data processing methods are presented in the “Methods” section. The observations are reported and discussed in the following section.

## Results and discussions

### Sound recordings

**Figure 1:**
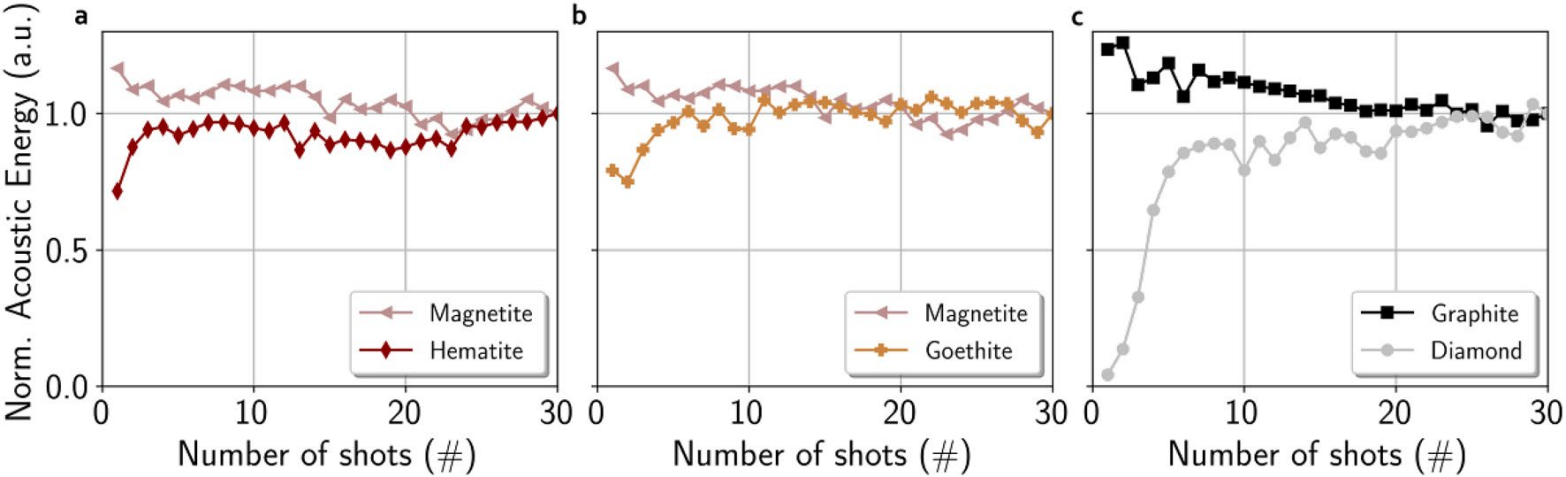
Evolution of the laser-induced acoustic energy over a burst of 30 LIBS shots on three couples of minerals: (**a**) hematite and magnetite, (**b**) goethite and magnetite and (**c**) graphite and diamond. For each target, the acoustic energy is normalized by the value of the last shot of the burst. The increase of the acoustic energy over the first 5 shots which is observed for hematite, goethite and diamond is discussed in the following sections, with regard to a change in laser-matter interaction for these specific targets.

The shot-to-shot evolution of the acoustic energy for bursts of 30 shots for the various minerals is presented in Fig. 1. For hematite and goethite, it is compared with the shot-to-shot evolution recorded on magnetite ($\hbox {Fe}_3\hbox {O}_4$), which is known not to be affected by any laser-induced phase transition^11^. Diamond is compared with the shot-to-shot acoustic signal from the graphite sample. Both magnetite and graphite show an expected monotonous decrease of the acoustic energy: the formation of an ablation cavity reduces the laser-matter interaction and thus the acoustic energy^5,6^. As graphite is softer than magnetite (Vickers hardness of 23 and 767 respectively), its decrease rate is larger compared to magnetite. On the other hand, hematite, goethite and diamond exhibit a sharp increase of the acoustic energy during the $\sim 5$ first shots, about 25% for both iron oxy(hydroxi)des and by a factor of 5 for diamond.

This increase in the acoustic energy for these three targets may be indicative of a change in the laser-matter interaction over the first 5 shots and, therefore, of a potential transformation of the target induced by the laser. Hence, craters obtained from 1 to 5 shots on a crystal of hematite, goethite and diamond are further characterized by Raman spectroscopy and SEM to elucidate the cause of the increasing acoustic energy recorded by the microphone data.

### SEM imaging and Raman analysis of LIBS craters

#### LIBS craters in hematite

**Figure 2:**
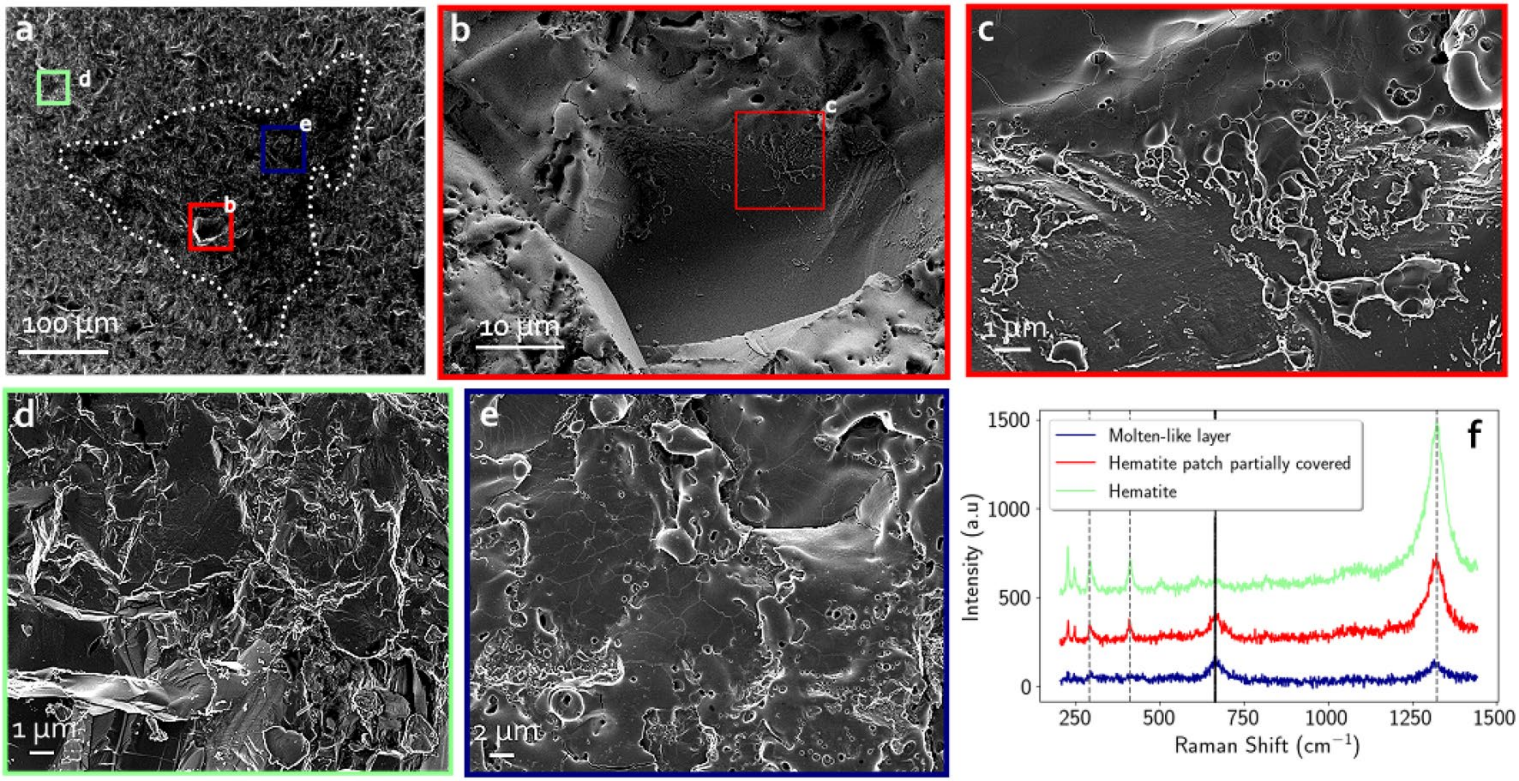
SEM images of the 1 shot deep crater in hematite. (**a**) Overview of the crater where its borders are indicated by a dotted line. Locations of the three zoomed images are represented by color squares. (**b**, **c**) Two subsequent zooms on a hematite patch partially covered by molten ejecta. (**d**) Zoom on the pristine hematite outside of the laser impacted zone. (**e**) Zoom on the molten-like layer (**f**) Raman spectra acquired in the color-associated area inside the crater. The vertical dashed grey lines highlight the main hematite bands at $291\ \hbox {cm}^{-1}$, $411\ \hbox {cm}^{-1}$, and $1322\ \hbox {cm}^{-1}$. The vertical solid black line highlights the main magnetite band at $664\ \hbox {cm}^{-1}$.

SEM images of the crater resulting from 1 laser shot are shown in Fig. 2. The crater is identified by a transition between a lighter toned surface, corresponding to the initial pristine hematite, to a darker surface that appears to have been texturally transformed. The close-up view of the hematite outside the impact zone (Fig. 2d) shows that the poly-crystalline mineral has a microtexture with grain boundaries and/or cleavages yielding a micrometer-scale roughness. The crater rim is irregular and the transition from the unaltered zone to the targeted area is diffuse, even at a finer scale. The LIBS crater floor is composed of two different microtextures. A few patches of pristine hematite are still observed in the inner part (Fig. 2b) but are partially covered by ejecta with a smooth texture similar to molten metal (Fig. 2c). These patches have a Raman signal consistent with hematite (red spectrum in Fig. 2f showing characteristic bands at $291\ \hbox {cm}^{-1}$, $411\ \hbox {cm}^{-1}$, and $1322\ \hbox {cm}^{-1}$) but with a rising band at $664\ \hbox {cm}^{-1}$ which is characteristic of magnetite.

In contrast, most of the crater floor is composed of a rough, cracked layer of molten metal that appears to slightly cover the pristine hematite because it has similar roughness (blue insert in Fig. 2a and e). This zone has a typical Raman signature of magnetite with a peak at $664\ \hbox {cm}^{-1}$. The hematite peak at $1322\ \hbox {cm}^{-1}$ is still present but has a lower relative intensity compared to the magnetite peak observed in the partially transformed zone. It shows that this layer of molten metal may not entirely cover the Raman analytical spot and that some hematite is still present, as detected by Raman (the Raman analytical spot is $\sim$1 - $2\ \upmu \mathrm{m}$, see the “Methods” section for detailed information on the Raman analysis).

**Figure 3:**
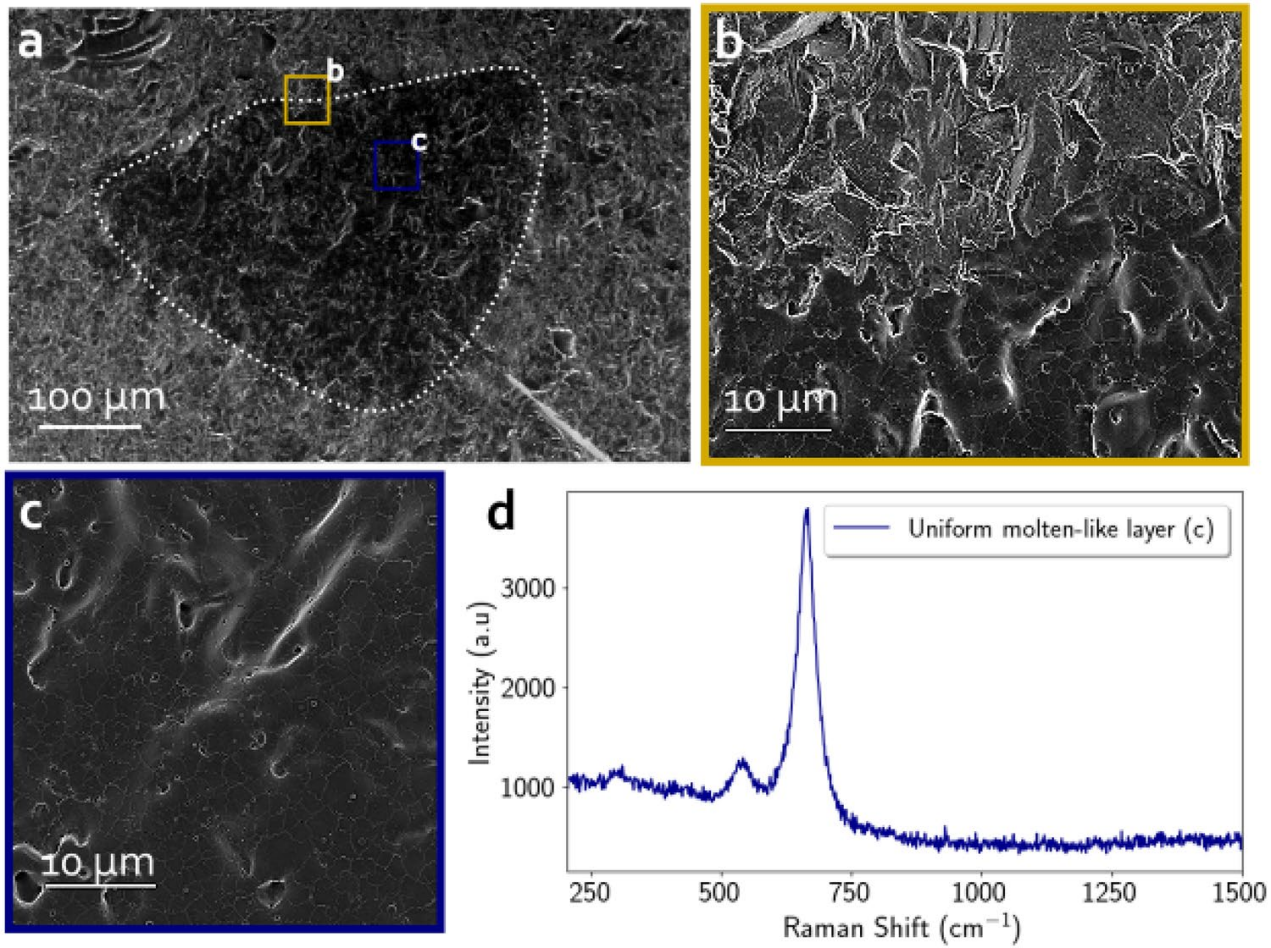
SEM images of the 5 shot deep crater on hematite. (**a**) Overview of the crater where its borders are visible. Locations of the two zooms depicted are represented by color squares. (**b**) Close-up view of the crater border showing a sharp transition. (**c**) Zoom on the uniform molten-like layer composing the crater floor. (**d**) Raman spectrum of pure magnetite acquired in the molten-like layer displayed in (**c**).

Remaining pristine hematite patches in the crater floor progressively disappear with an increasing number of laser shots. Figure 3 displays SEM images for the laser-induced crater resulting from 5 cumulative shots at the same location. The transition between the pristine hematite and the inner crater is sharp and clearly visible (see dashed line in Fig. 3a and close-up view in Fig. 3b). No more hematite patches are detected inside the crater and its floor is now flat and totally covered by the molten-like material. All the roughness has been covered uniformly by this molten layer but lots of cracks can be observed on its surface, likely due to thermal contraction during a fast cooling. All Raman spectra measured inside the crater perfectly matches with magnetite as it displays bands at $531\ \hbox {cm}^{-1}$ and $664\ \hbox {cm}^{-1}$ (see Fig. 3d). The hematite bands, previously observed in the one shot crater, are here totally absent. The molten-like layer of the crater floor after 5 shots is thick enough to completely overlay the hematite and to smooth the roughness of the initial hematite mineral. These SEM observations, supported by single point Raman spectra, indicate that the transition from the hematite to the magnetite in the ablation zone was completely achieved within the first 5 shots.

**Figure 4:**
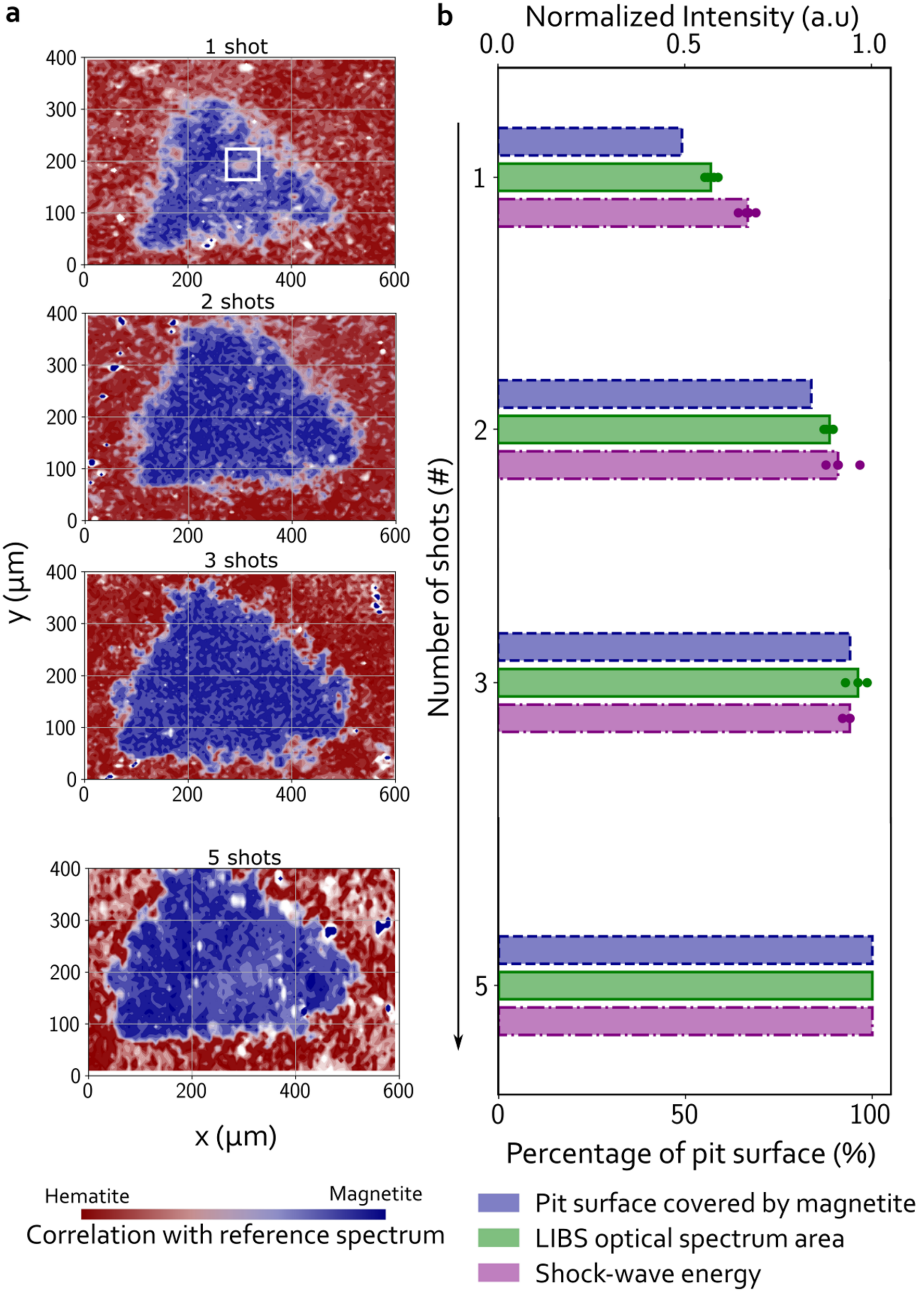
(**a**) Raman mapping of laser-induced craters on hematite resulting from 1, 2, 3 and 5 shots. Color scale indicates the correlation of the material with a reference hematite Raman spectrum (red tones) and with a reference magnetite Raman spectrum (blue tones). The white square in the 1 shot crater highlights the hematite patch identified in Fig. 2. (**b**) Comparison between the percentage of the crater surface covered by magnetite (blue bars), the LIBS optical spectrum area in the UV range (green) and shock-wave energy (purple). Values for the LIBS optical spectrum area and the shock-wave energy are normalized by the values of the 5^th^ shot. Colored circles correspond to the value of all the measurements performed for a given number of shot (for example, for shot 2, each point represents the value for the 2^th^ shot of bursts with 2 shots or more). The heights of the bars are a median among these points. The percentage of the crater covered by magnetite is computed as the number of pixels that have more than 60% of correlation with the reference magnetite Raman spectrum over the total number of pixels that compose the crater.

To further characterize the transition occurring during the first 5 shots, Raman hyperspectral mapping was performed on craters excavated with 1, 2, 3 and 5 LIBS shots in the hematite crystal. A percentage of correlation with the Raman spectrum of magnetite and hematite is extracted for each pixel/spectrum and represented in the maps in Fig. 4a (see section “Methods” for the detail of the processing leading to these maps). These maps confirm the SEM observations and the single point Raman analysis discussed above. The 1 shot crater measures $\sim 420 \ \upmu \mathrm{m}$ in length along its horizontal axis with some remaining micrometric patches of hematite inside the pit (see the reddish feature in the white square the corresponds to the hematite patch highlighted with SEM in Fig. 2b). Considering the entire surface of the 1 shot crater, only 50% of the surface shows a predominantly (i.e. correlation of more than 60%) magnetite signal. Therefore, firing only one laser pulse on a hematite crystal is therefore sufficient to transform the major part of the focused area into a molten-like layer that has a Raman signal partly consistent with magnetite. The crater made with 2 LIBS shots is $\sim 430\ \, \upmu \mathrm{m}$ long and has fewer hematite patches than the single shot crater. The magnetite Raman signal is more homogeneous: 83% of the crater surface is predominantly magnetite. After 3 shots, the crater boundaries are sharp and the crater reached its maximal size of $\sim 440\ \, \upmu \mathrm{m}$ . Hematite has almost completely disappeared inside the smoothed and flatten crater: 94% of the 3 shot crater is covered by the molten-like magnetite signal material. Ultimately, 100% for the 5 shot crater is covered with magnetite. Therefore, the transition between hematite and magnetite fully occurred during the first 5 shots.

The comparison between the magnetite occurrence from Raman maps within the crater, the acoustic energy and the LIBS optical spectrum area is shown in Fig. 4b. Both the acoustic energy and the LIBS optical signal increase over the 5 first shots on the hematite crystal. Both observations tend to support that the laser-matter interaction increased over the 5 first shots due to the transition of hematite into magnetite by melting followed by recrystallization.

#### LIBS craters in goethite

**Figure 5:**
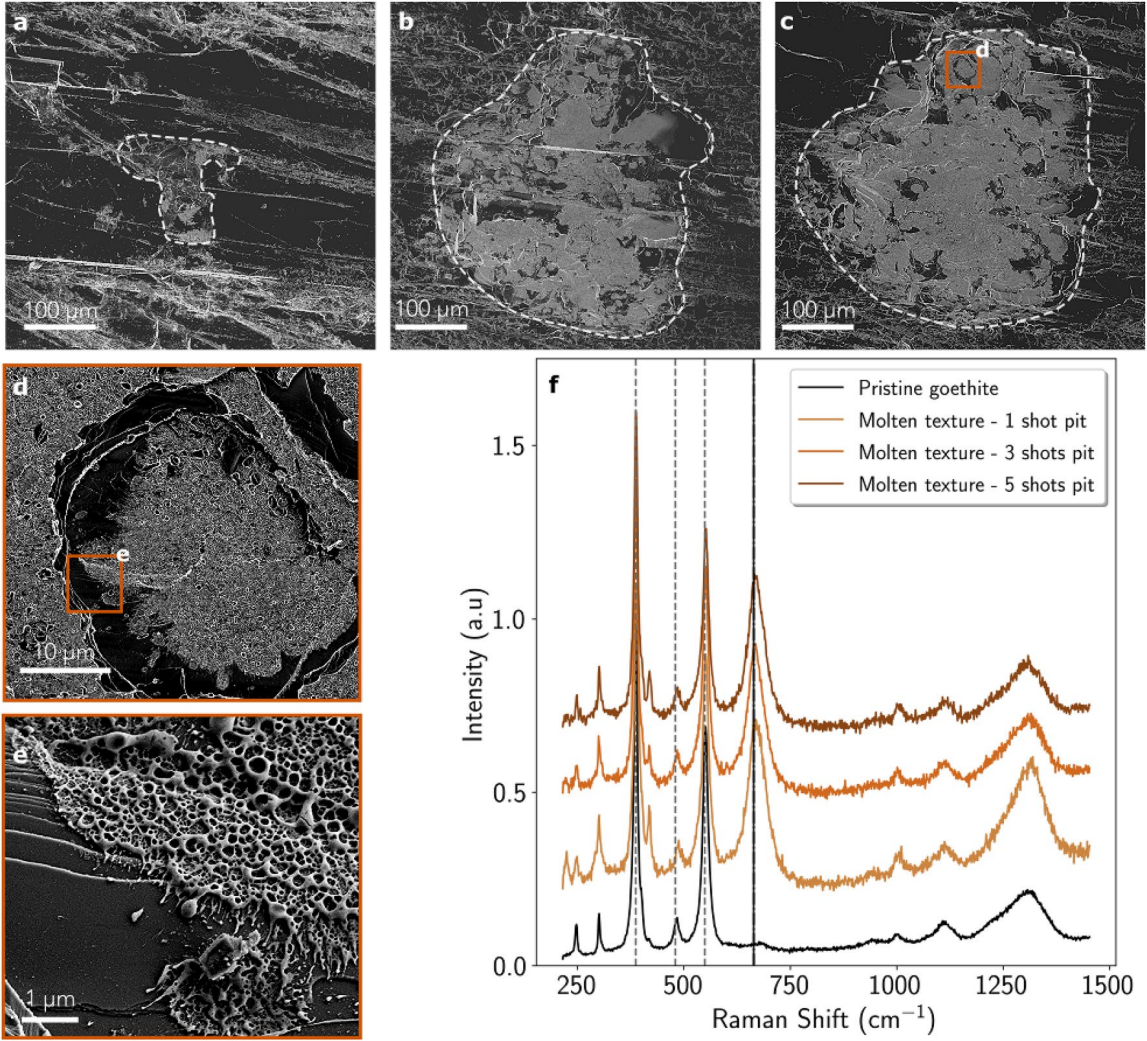
SEM images of the 1 (**a**), 3 (**b**) and 5 shot (**c**) deep craters on goethite. The crater borders are highlighted by the dashed grey lines. (**d**, **e**) Two subsequent zooms in the 5 shot deep crater that exhibits both the molten alveolar texture and the smooth pristine goethite. (**f**) Four Raman spectra acquired on a pristine goethite spot (black spectrum) and on the molten alveolar texture (brown spectra) seen in the three craters presented in (**a**–**c**). The vertical dashed grey lines highlight the main goethite bands at $387\ \hbox {cm}^{-1}$, $480\ \hbox {cm}^{-1}$, and $549\ \hbox {cm}^{-1}$. The vertical solid black line highlights the main magnetite band at $664\ \hbox {cm}^{-1}$.

Craters obtained with 1, 3 and 5 shots on goethite were investigated by SEM as illustrated in Fig. 5. Whereas the pristine goethite has a flat surface with some cracks and steps, the crater floor generated by the laser is recognized thanks to its light toned cover (delimited by the grey lines in Fig. 5a–c). The area covered by the light toned texture increases with the number of shots fired at the same location. Inside all the craters, some dark toned patches are present, but they are less numerous in the 5 shot crater compared to the 3 shot crater. The close-up views of the 5 shot crater (Fig. 5d,e) highlight the two microtextures seen in the pit. On the one hand, the darker patches are consistent with the initial pristine goethite, which is confirmed by their Raman signal (see black curve in Fig. 5f), with characteristic peaks at $387\ \hbox {cm}^{-1}$, $480\ \hbox {cm}^{-1}$, and $549\ \hbox {cm}^{-1}$. On the other hand, the light toned texture that covers most of the crater surface looks like molten metal, as seen in the hematite craters, but it is much more alveolar, i.e. lots of cavities, and it looks more surficial than the molten layer observed on hematite craters. Goethite seems to outcrop right below this alveolar texture. This light tone alveolar patch has a Raman signal which is still consistent with goethite but with an additional peak at $664\ \hbox {cm}^{-1}$, consistent with magnetite (see brown curves in Fig. 5f). The magnetite signal may come from this molten alveolar patch, whereas the goethite peaks may originates from pristine goethite lying beneath but sampled by the Raman laser beam through the holes in the alveolar patch. Although the area covered by this molten texture increases with the number of shots, the microtextural aspect and the Raman signal of this patch do not evolve with the number of shots meaning that the molten alveolar patch does not thicken with an increasing shot number, contrary to what was observed on hematite.

These observations suggest that firing a pulsed laser on a goethite crystal induces a phase transformation on the goethite into a thin layer composed of magnetite inside the laser-induced crater. As for hematite, the increase of the area covered by the transitioned phase is correlated with an increase of the laser-induced acoustic signal recorded during the first shots of the laser burst.

#### LIBS craters in diamond

**Figure 6:**
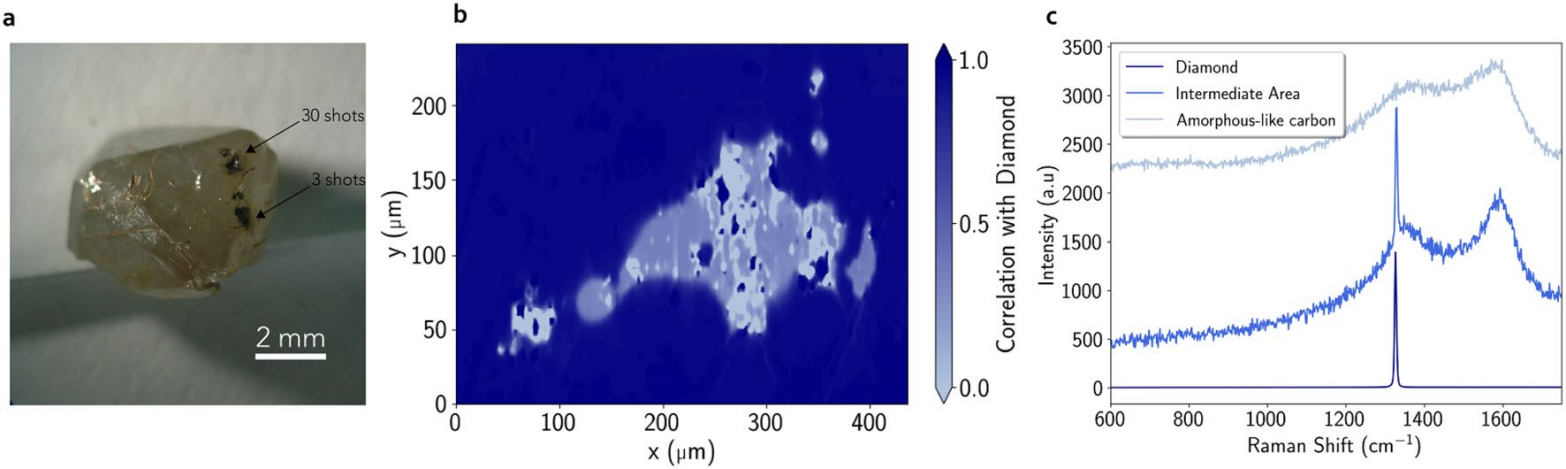
Observations of the LIBS craters performed in a diamond. (**a**) Image of the diamond used in this study. Two LIBS craters are visible as black spots, one made with 30 shots and one with 3 shots. (**b**) Raman mapping of the 3 shot deep crater. The color scale indicates the correlation with the out-of-crater diamond Raman spectrum. (**c**) Three spectra extracted from the map showing the transition from pure crystalline diamond to amorphous carbon inside the LIBS crater.

In the case of diamond, during the 5 first shots, a very small area is ablated compared to the ablations on hematite or goethite. The 3 shot LIBS crater for diamond has an irregular shape (see Fig. 6a,b) and is only $\sim 350\ \upmu \mathrm{m}$ long on its longer axis compared to $440\ \upmu \mathrm{m}$ on hematite for the same number of shots. After 5 shots, the crater floor is progressively covered by a dark layer with a carbonized aspect (see Fig. 6b, c for a LIBS crater made in diamond with three shots). Whereas the sharp and intense $1332\ \hbox {cm}^{-1}$ Raman peak for diamond is observed out of the LIBS crater (dark blue spectrum in Fig. 6c), its intensity strongly decreases and locally disappears in the LIBS crater. On the dark material in the crater, the Raman spectrum exhibits two large bands centered at about $1345\ \hbox {cm}^{-1}$ (D-band) and $1600\ \hbox {cm}^{-1}$ (G-band) (lighter blue spectra in Fig. 6b). Such spectra on the black material are consistent with amorphous-like carbon or locally very disordered graphitic carbon^16,17^, showing that the laser beam has locally transformed the crystalline structure of diamond into amorphous-like carbon in the crater.

### Interpretation

#### The case of iron oxides

The observations of the laser-induced craters show that the laser has strongly affected the mineralogical structure of the hematite and goethite. Some experiments and existing models of temperature rise within hematite^11^ show that the laser is able to heat the material up to its melting point (1838 K) over a depth between 200 nm and $1\ \, \upmu \mathrm{m}$. In such a model, material in the crater remains transiently in a liquid state for hundreds of ns after the laser pulse. As the melting starts, a liquid-vapor equilibrium is established above the surface of the liquid, and therefore, vaporization starts^3,18^. Under Mars atmosphere, the melting temperature of both hematite and magnetite can be estimated using the temperature-pressure diagram calculated for the Fe-O system^19^. Assuming that the maximum oxygen partial pressure corresponds to the maximum possible $\hbox {O}_2$ released by the breakdown of all the $\hbox {CO}_2$ molecules of the martian atmosphere in the LIBS plasma, the melting temperature is around 1680 K. As suggested by SEM analyses and a first-order theoretical approach^11^, such a temperature was likely exceeded in the LIBS crater. In any case, the quench of the melt leads to the crystallization of magnetite and not hematite as predicted by the temperature-pressure diagram calculated for the Fe–O system^19^. This magnetite remains stable within the layer covering the LIBS crater and does not back-transform into hematite as observed by Raman spectroscopy, even after a single LIBS shot. The molten layer is very thin at the surface of hematite, about 200 nm^11^. The very fast cooling favors the crystallization of submicrometer-sized magnetite crystals. Interestingly, the thickness of the magnetite layer at the surface of hematite is large enough to explain why the Raman only probes magnetite and not the underlying hematite. Indeed, the high absorption optical coefficient of magnetite at 532 nm^20^, i.e. about $2 \times 10^5\ \, \hbox {cm}^{-1}$, yields an optical penetration of $\sim 50\ \, \upmu \mathrm{m}$ which is lower than the actual thickness observed for the magnetite layer.

Therefore, one could wonder why an increase of the laser-induced acoustic signal is observed on hematite and goethite, which both suffer a phase transition, whereas such an increase is not observed when firing the laser on a pure magnetite crystal (see Fig. 1a,b). Laser-induced acoustic signal is shown to be an indicator of the ablation process and therefore, an indicator of the strength of the coupling between the laser and the sample^21^. For the target, both its thermal and optical properties govern how efficient is the laser-matter interaction. The optical penetration depth $\delta _{\text {opt}}$ (defined as the inverse of the absorption coefficient of the sample at the laser wavelength) gives the depth over which the laser radiation is absorbed within the sample. The shorter the optical penetration depth is, the more energy per unit of volume is available for ablation. On the other hand, this absorbed laser energy is converted into heat and penetrates into the sample over the thermal penetration depth, $\delta _{\text {th}}$, which is a function of the thermal diffusivity of the material and the laser pulse duration. The larger the thermal diffusivity is (and then the thermal penetration depth), the faster the heat is dissipated away from the optical absorption area^22^. Thus, ablation is less efficient for long penetration depth. It was also shown that the surface aspect is a key parameter that controls the laser-matter interaction: steep walls receive a lower irradiance compare to planar horizontal surface^5^.

After only one laser pulse on the hematite and goethite, the crater floor is partially transformed into the molten-like magnetite. Therefore, the subsequent pulses are fired on this new phase that has different properties compared to the initial pristine mineral (see Table 1a). Indeed, magnetite has both its thermal and optical penetration depths shorter than hematite and goethite. Therefore, the energy of the next laser pulse is absorbed within a smaller depth and its heat is less dissipated: it leads to a more efficient ablation on the transformed magnetite layer, and consequently a stronger shock-wave than on the pristine mineral.

Furthermore, in the case of the hematite, the roughness of the initial pristine material explains that, in some part of the targeted surface (hematite patch in a small cavity displayed in Fig. 2) the laser-matter interaction is weaker due to a steeper surface, hence the melting point is not reached locally. Moreover, as the laser has a Gaussian shape for the energy profile, the irradiance is lower on edges. Therefore, on the crater rims, the energy transferred to the material is not high enough to allow a complete hematite melting. This likely explains that the crater rims are not well defined for the first shot. The second shot ablates the previously melted and recrystallized material, and it induces heating of the underlying hematite by heat transfer. Therefore, the layer of molten material thickens. The new pulse also affects and transforms the remaining hematite patches. Before it recrystallizes, the liquid metal may spread on the surface^23^ and progressively covers the asperities of the initial hematite (this iron melt stays liquid during 500 ns after the laser pulse^11^). Consequently, over the 5 first shots on hematite, the increase of the magnetite coverage in the crater explains the increase of the laser-induced acoustic signal. Similarly, the increase of the laser-matter interaction over the five first shots leads to a more luminous plasma and a stronger LIBS optical spectrum.

**Figure 7:**
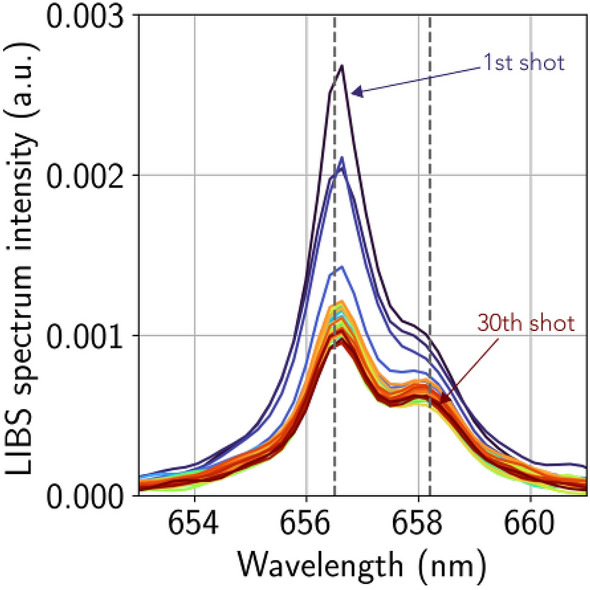
Evolution of the LIBS hydrogen line (656.6 nm) observed on goethite as a function of the number of shots, from blue (1st shot) to dark red (30th shot). The peak at $\sim 658 \, \upmu \mathrm{m}$ is a carbon peak doublet from the breakdown of the $\hbox {CO}_2$ molecule of the atmosphere. Spectra are normalized to the mean intensity of the total spectrum.

For goethite, as for hematite, the increase of the laser-induced acoustic signal over the first shots is likely due to a similar process. This may be explained, in the same way, by a large optical penetration depth for goethite (see Table 1a, despite the large uncertainty of this value for goethite). However, the short thermal penetration depth for goethite shows that, as the heat does not evacuate from the absorption zone, the temperature rise in the goethite is more confined. Therefore, goethite may melt over a very thin layer compared to hematite, which may explain the low thickness aspect of the molten-like magnetite layer observed by SEM on the goethite crater floors (Fig. 5). The clear pristine goethite patches observed in the craters may be caused by excavation of layered-material due to the strength of the blast. Goethite is a hydrated mineral and the hydrogen LIBS peak (Fig. 7) shows sharp decline over the 4 first shots on the goethite, before its intensity stabilizes for the following shots. These details support the hypothesis that goethite has transformed to magnetite and thus, has dehydrated. Moreover, the alveolar microtextural aspect of the magnetite layer seen on the goethite craters but not on hematite, may be generated by the presence of outgassing water during the vaporization process.

Finally, as magnetite recrystalizes into magnetite after a laser shot^11^, there is no change in physical properties in the ablation zone. As a consequence there is no large change in laser-matter interaction from one shot to another and therefore, no increase of the acoustic energy.

**Table 1:** Physical properties of the phases analyzed.

(a) Optical and thermal properties of hematite, goethite and magnetite used to characterize the laser matter interaction. The optical penetration depth for goethite is taken at 750 nm, the closest value available in the literature^28^.					
Mineral	Formula	$\delta _{\text {th}}$ (nm)	$\delta _{\text {opt}}$ (nm)	Melting point (K)	References
Hematite	$\hbox {Fe}_2\hbox {O}_3$	140	7700	1838	^24–26^
Goethite	FeO(OH)	67	846	623	^24,27,28^
Magnetite	$\hbox {Fe}_3\hbox {O}_4$	92	229	1811	^24–26^

(b) Optical and thermal penetration depth for diamond, graphite and amorphous carbon. For graphite, separate entries are given for the thermal penetration parallel (par.) and perpendicular (perp.) to the graphene planes. (*) The optical penetration depth could not be found for amorphous carbon. Considering its visual aspect, we assume that its optical properties are similar to those of graphite.					
Phase	$\delta _{\text {th}}$ (nm)	$\delta _{\text {opt}}$ (nm)	References		
Diamond	2363	between 1.3 $\times 10^7$ and $2.2 \times 10^8$	^29^		
Graphite	2557 (par.) 140 (perp.)	41	^29,30^		
Amorphous	141	41(*)	^31^		

#### The case of diamond

As with iron oxides, the amorphization seen in diamond craters explains the sudden increase in the LIBS acoustic signal amplitude over the first shots, as it induces a change of the physical properties within the ablation zone. Table 1b lists the physical properties of diamond, graphite and amorphous carbon. It is noticed that diamond is almost transparent to the LIBS infrared laser: its optical penetration depth is more than one million times greater than for amorphous carbon. The coupling of the laser on pure diamond is therefore theoretically very weak to null. This explains the very low intensity of the acoustic signal for the first shot. However, this natural diamond has some impurities and defects on which the laser can couple more easily. Therefore, the laser energy is absorbed locally which triggers a weak ablation and local transformation of diamond into amorphous-like carbon. This amorphous carbon absorbs much more the laser radiation, increasing the coupling conditions for the next shot. The more the firing sequence progresses, the more the amorphized zone extends and the more the coupling improves. This increase in coupling during the first shots implies an increase in the amplitude of the acoustic signal.

On the other hand, no remarkable phenomenon is observed on the acoustic signal recorded from graphite (Fig. 1c) whereas the work of Fau et al.^11^ shows an amorphization of the graphite after a burst of 30 laser shots. Thermal properties of graphite are anisotropic due to its structure as a stack of graphene planes (see the two thermal penetration depths over the two directions in Table 1b) while the properties of amorphous-like carbon are isotropic. One would expect an increase in coupling with amorphization of graphite because graphite dissipates heat more efficiently in its parallel plane than amorphous carbon. This is not seen in the acoustic data. One explanation would be that the loss of energy within the graphene planes which are perpendicular to the laser axis in our experiments may be negligible. In addition, right after the first laser shots the planar and stacking structure of initial graphite is likely locally destroyed within the LIBS crater yielding more isotropic thermal properties. Last, the acoustic increase that may be induced by this modification of thermal penetration depth, may be hidden by the decrease of the signal due to formation of a deep crater in this low hardness material^6^. For large number of shots, the global heating of the target may also be responsible of progressive changes in the target physical properties (thermal, optical and dielectric). Therefore, it may contribute to progressive loss in laser-matter coupling.

## Implications and perspectives for Mars targets analysis

Laser ablation transfers a huge energy density into the targeted material and this study showed that it locally affects the mineralogical structure of three of the samples analyzed: hematite, goethite and diamond. While this phenomenon was already described, especially on hematite^11^, the present study shows that it can also be instantaneously and remotely recorded by acoustic data. For the minerals experiencing a laser-induced phase transition, the laser-induced acoustic signal shows an increase of its amplitude over the first shots. It correlates with the formation of a new phase within the ablation cavity: magnetite for hematite and goethite, and amorphous-like carbon or locally very disordered graphitic carbon for diamond. The increase in the acoustic amplitude is explained by a change in the optical and/or thermal properties of the samples, as it is transformed by the laser. It consequently modifies the laser-matter interaction and then the strength of the shock-wave.

Although diamond is a less relevant target for Martian surface exploration, its study allows us to better understand the influence of the physical properties of the samples that drive the laser-induced acoustic signal, with a simple example. In particular, it is shown that the optical absorption coefficient plays a major role in the amplitude of the acoustic signal. Indeed, this is the first time that the physical properties of a sample (thermal and optical penetrations depths) are used to explain the behavior of the acoustic signal. More generally, this study shows how the microphone is an aid for mineralogy through the signatures of the physical properties of minerals.

On the other hand, the acoustic detection of phase transition on iron oxides has a major implication for the exploration of the Mars surface chemistry with LIBS. In Gale crater, almost pure iron oxide nodules are observed with the LIBS experiment of ChemCam. However, LIBS on Mars gives limited information on oxygen, as the oxygen signal in the LIBS spectrum originates from both the sample and the breakdown of the atmospheric carbon dioxide^32^. Thus, LIBS cannot detect the stoichiometric variations between the compositions of various iron oxides. In addition, LIBS cannot distinguish the degree of oxidation of these iron oxides based on the LIBS active spectrum, and thus distinguish between magnetite and hematite^33^. On Perseverance, the SuperCam instrument combines a LIBS experiment and a microphone. Given the results of this study, the observation of an increase in the LIBS acoustic signal amplitude on a pure iron oxide target would indicate the likely presence of hematite or goethite. Furthermore, the observation of the hydrogen line in the LIBS spectrum can help concluding between goethite and hematite. Of course, this acoustic method will be complementary to the other mineralogical investigations provided by SuperCam, e.g. visible and near-infrared reflectance spectroscopy. The Raman signal from these opaque iron oxides is very weak, and so, SuperCam does not have a chance to detect them with time-resolved Raman spectroscopy.

As for the goethite, this is the first observation of a mineral being dehydrated by LIBS. Therefore, one could wonder how it could be extrapolated to other hydrated minerals relevant to Mars. Previous experiments to track laser-induced dehydration on hydrated samples led to inconclusive results, except for gypsum for which no significant dehydration was observed^34^. Indeed, such a mineral has a large optical penetration depth and therefore, is less likely to experience a sharp temperature rise, which may prevent any phase transition and acoustic effects. Previous observations of LIBS craters on some low optical absorption hydrated samples, like gypsum or hydromagnesite, with Raman spectroscopy^11^ did not show any apparent dehydration or structural modification, at least over the entire volume excited. Indeed, for these kinds of mineral, the Raman signal comes from a relatively large volume and may not be fully representative of the very first nanometers at the surface. Consequently, more experiments on hydrated minerals with a different analytical strategy are needed to highlight the specific cases where laser-induced dehydration occurs, and if it can be systematically tracked with acoustic data.

As the laser-induced breakdown spectroscopy becomes a new standard for planetary exploration^35^, microphone may become a critical add-on to such instruments to provide key information on the physical properties of the samples analyzed.

## Methods

### LIBS and acoustic test bench

 The LIBS capability of this setup used the ChemCam Mast-Unit Engineering and Qualification Model that deposits about 10 mJ on a target through a 5 ns infrared pulse (1067 nm). The laser was fired at a frequency of 3 Hz. The Mast-Unit was coupled with the ChemCam Body-Unit Engineering Model that includes three spectrometers collecting the radiations from the plasma over the UV (240.1 nm to 342.2 nm), the violet (382.1 nm to 469.3 nm) and the visible plus near infrared (VNIR, 474 nm to 906 nm). The laser beam was redirected into a vacuum chamber that contains the samples. The chamber was filled with a controlled Mars atmosphere (95.7% of $\hbox {CO}_2$, 2.7% of $\hbox {N}_2$ and 1.6% of Ar) at a pressure of 6 mbar. The chamber includes a microphone which points toward the targets. It is a SuperCam microphone, Knowles Electret condenser microphone, model EK-23132. The sensitivity was $22.4\ \hbox {m V Pa}^{-1}$. The microphone signal was sampled at a frequency 200 kHz and it recorded the LIBS burst continuously from the first shot to the last one. The acoustic energy, in $\hbox {Pa}^2$.s, is computed as the square value of the acoustic waveform over the compression phase. The LIBS spectra were processed with the standard pipeline used for Chemcam^36^ (de-noising, continuum removal and wavelength calibration). Due to an idiosyncrasy of the telescope of this setup, the ablation crater is composed of two lobes, which can be seen in Fig. 6a. Only the biggest one is considered in this study, as it represents more than 80% of the total surface ablated.

### Raman spectroscopy

All samples were first analyzed using a continuous-wave Raman microspectrometer Renishaw InVia Reflex for point analyses and Raman mapping when needed. Measurements were performed using a green 532 nm solid-state laser focused on the sample through a Leica DM2500 microscope with a long-working distance 50 × objective (NA = 0.55). This configuration yielded a horizontal resolution of $\sim$1–$2\ \, \upmu \mathrm{m}$ for a laser power delivered at the sample surface set at less than 1 mW using neutral density filters to prevent irreversible thermal damages. This corresponds to a laser irradiance in the range of $\sim$0.3–$1.3\ \, \hbox {GWm}^{-2}$. All measurements were performed with a circularly polarized laser using a $\tfrac{1}{4}$ wavelength plate placed before the microscope in order to minimize polarization effects. The Raman signal was dispersed by a grating with 2400 lines/mm and the signal was analyzed with a RENCAM CCD detector. For Raman mapping and the acquisition of hyperspectral maps, the sample was moved with an appropriate step size using a XYZ Renishaw motorized stage. Laser focus was optimized by correcting topographic variation prior to analysis. For the maps presented in Fig. 4, a Raman spectrum was acquired every $2.6\ \, \upmu \mathrm{m}$ in both axis. Each spectrum is compared with both a pure hematite and pure magnetite Raman spectrum with a direct classical least square method. A percentage of correlation with hematite and magnetite is computed and is used to draw the maps. For Fig. 6, the method and the resolution are the same. More about Raman mapping can be found in Bernard et al.^37^. All measurements were performed at room temperature and spectra were recorded directly on the raw samples without any preparation.

### Scanning electron microscopy analysis

Scanning electron microscopy (SEM) analyses were performed using a Zeiss Ultra 55 field emission gun SEM. Secondary electron images were acquired using an in-Lens detector (for nanotopography of the sample) at an accelerating voltage of 5 kV, a working distance of $\sim 3\ \, \upmu \mathrm{m}$ and a $60\ \, \upmu \mathrm{m}$ aperture at high current.

### Samples

 Minerals were obtained from the Collection de Minéralogie at Sorbonne Université (Paris, France). These are natural single crystals. The provenances are: hematite (Elba Island, Italy), magnetite (Chester, Vermont, USA), goethite (Restormel, Cornwall, UK), graphite (Madagascar) and diamond (Kasai Province, Democratic Republic of Congo).
